# The role of cyclin D1 and Ki‐67 in the development and prognostication of thin melanoma

**DOI:** 10.1111/his.14139

**Published:** 2020-07-04

**Authors:** Corina Kaufmann, Werner Kempf, Joanna Mangana, Phil Cheng, Michael Emberger, Roland Lang, Andreas K Kaiser, Evelyn Lattmann, Mitchell Levesque, Reinhard Dummer, Peter Koelblinger

**Affiliations:** ^1^ Department of Dermatology University Hospital Zurich Zurich Switzerland; ^2^ University of Zurich Zurich Switzerland; ^3^ Kempf and Pfaltz Histological Diagnostics Zurich Switzerland; ^4^ Private Pathological Laboratory Salzburg Austria; ^5^ Department of Dermatology and Allergology Paracelsus Medical University Salzburg Austria; ^6^ Department of Clinical Psychology Christian‐Doppler‐Klinik Paracelsus Medical University Salzburg Austria

**Keywords:** biomarker, cyclin D1, immunohistochemistry, Ki‐67, thin melanoma

## Abstract

**Aims:**

Despite their low individual metastatic potential, thin melanomas (≤1 mm Breslow thickness) contribute significantly to melanoma mortality overall. Therefore, identification of prognostic biomarkers is particularly important in this subgroup of melanoma. Prompted by preclinical results, we investigated cyclin D1 protein and Ki‐67 expression in *in‐situ*, metastatic and non‐metastatic thin melanomas.

**Methods and results:**

Immunohistochemistry was performed on 112 melanoma specimens, comprising 22 *in situ*, 48 non‐metastatic and 42 metastatic thin melanomas. Overall, epidermal and dermal cyclin D1 and Ki‐67 expression were semiquantitatively evaluated by three independent investigators and compared between groups. Epidermal Ki‐67 expression did not differ statistically in *in‐situ* and invasive melanoma (*P = *0.7). Epidermal cyclin D1 expression was significantly higher in thin invasive than in *in‐situ* melanoma (*P = *0.003). No difference was found in cyclin D1 expression between metastatic and non‐metastatic invasive tumours. Metastatic and non‐metastatic thin melanomas did not show significant differences in epidermal expression of Ki‐67 and cyclin D1 (*P = *0.148 and *P = *0.611, respectively). In contrast, strong dermal expression of Ki‐67 was more frequent in metastatic than non‐metastatic samples (28.6 versus 8.3%, respectively, *P = *0.001). The prognostic value of dermal Ki‐67 expression was confirmed by multivariate analysis (*P = *0.047).

**Conclusion:**

We found an increased expression of cyclin D1 in invasive thin melanomas compared to *in‐situ* melanomas, which supports a potential role of this protein in early invasion in melanoma, as suggested by preclinical findings. Moreover, our results confirm that high dermal Ki‐67 expression is associated with an increased risk of development of metastasis in thin melanoma and could possibly serve as a prognostic biomarker in clinical practice, especially if combined with additional methods.

## Introduction

The incidence of invasive cutaneous melanoma has been steadily increasing in countries of the western world for more than five decades.^1^ Owing to improved skin cancer screening and diagnostic techniques, the majority of cutaneous primary melanomas is diagnosed at an *in‐situ* or early invasive stage. Despite the low individual metastatic potential of these tumours, the subgroup of thin invasive melanomas (up to 1 mm in Breslow thickness) accounts for the highest absolute number of melanoma‐related deaths of all four T subcategories as defined by the American Joint Committee on Cancer (AJCC) staging system.^2^, ^3^, ^4^ Hence, understanding of the early cellular and genetic mechanisms that enable melanoma cells to breach the basement membrane and acquire invasive potential is crucial to allow for early identification of selected patients at increased risk of developing metastatic disease. With regard to genetics, melanoma development has been described as a distinct stepwise process with a successive evolution from unequivocally benign to intermediate, early malignant (*in situ*) and invasive malignant melanocytic lesions based on distinct alterations, including initial BRAF V600E mutations, among others.^5^ The diverse cellular consequences of these oncogenic mutations that confer tumour growth, invasion and metastatic potential constitute the well‐known hallmarks of cancer.^6^ One of these hallmarks is sustained proliferative signalling which, *in vivo*, can be depicted through detection of proliferation markers such as the Ki‐67 protein. This protein is expressed in the nuclei of cells in the G1, S and G2 phases of the cell cycle, as well as in mitosis.^7^ The functional significance of the Ki‐67 protein within the cell cycle remains to be fully elucidated. Important roles in cancer stem cell maintenance and organisation of the chromosome periphery during mitosis have been described. Clinically, increased Ki‐67 expression is a well‐known marker of poor prognosis in various malignancies such as breast cancer.^8^ In melanoma, its prognostic role was most clearly shown in thick primary tumours (Breslow> 1 mm).^9^, ^10^ In thin primary melanomas, particularly high dermal Ki‐67 expression has been associated with increased risk for metastasis development.^11^

Another potential marker of tumour cell proliferation is cyclin D1. This highly labile protein regulates G1/S transition in the cell cycle through interaction with cyclin‐dependent kinases which mediate subsequent phosphorylation of the retinoblastoma protein leading to increased proliferation, among other cellular changes.^12^, ^13^ The CCND1 gene encoding for cyclin D1 is an established oncogene amplified in a variety of tumours such as breast, lung or endometrial cancer.^12^ Increased expression of cyclin D1 has also been reported in up to 62% of primary melanomas, while being minimal in melanocytic naevi.^14^ The oncogenic effect of cyclin D1 overexpression or gene amplification is primarily attributed to its impact on tumour cell proliferation, but proliferation‐independent oncogenic mechanisms have also been proposed.^12^ These include effects of cyclin D1 on cellular migration and invasion, which were first described in macrophages.^15^ In this context, preclinical studies at our institution have shown that expression of cyclin D1 may confer early invasive properties on melanoma cells. As results concerning the prognostic significance of cyclin D1 expression in melanoma are inconsistent,^16^, ^17^, ^18^, ^19^ based on our preclinical data we hypothesised that cyclin D1 expression in primary melanoma may not only be a surrogate for cellular proliferation, but also for early invasive properties of melanoma cells. Therefore, the present study investigated the expression and correlation of cyclin D1 and Ki‐67 at different stages in the stepwise development of invasive melanoma. Both *in‐situ* and thin invasive primary melanomas were examined. Additionally, thin primary melanomas leading to the development of metastatic disease were specifically analysed in order to explore the potential role of cyclin D1 later in the process of invasion and metastasis development, hence re‐evaluating its potential as a prognostic marker.

## Materials and methods

### Patient Selection and Data Collection

The study was approved by both local ethics committees [Zurich (KEK‐ZH‐No. 647 and 800) and Salzburg (E‐No. 2252)] prior to collection of data and tissue specimens. Clinical information was stored and analysed after encryption.

The study cohort consisted of patients with *in‐situ* (MIS), metastatic (MTM) and non‐metastatic (NMTM) thin melanomas diagnosed during the period from 2008 and 2016 at two melanoma reference centres in Zurich and Salzburg. Disease stage was classified according to the 7th edition of AJCC staging system.^20^ Patients with multiple primaries, uveal or mucosal melanoma were excluded. Epidemiological, clinical and laboratory information were retrospectively collected from medical records. Multiple dermato‐pathological institutes in and around Zurich and an external collaborating pathology institute (Salzburg) served as sources for formalin‐fixed paraffin‐embedded (FFPE) samples of the respective primary tumours.

### Immunohistochemistry (IHC)

Four different analyses [including haematoxylin and eosin (H&E) stain] were conducted on 3‐μm‐thick FFPE sections. Processing comprised deparaffinisation in xylene and rehydration in decreasing ethanol concentrations, consecutive boiling for epitope retrieval in a 3‐in‐1 target retrieval solution (TRS6 or 9; Dako^®^, Agilent Technologies, Santa Clara, CA, USA) in a pressure cooker for a total of 40 min, followed by cooling for 20 min and rinsing with deionised water. Further staining was performed using the automated Dako^®^ Autostainer Plus platform (see Supporting information, Table S6). The following primary antibody clones were used: HMB‐45 clone HMB45 (M0634), Ki‐67 clone MIB 1 (M7240) and cyclin D1 clone EP12 (M3642), all manufactured by Dako.

### Immunoreactivity Scoring

Three independent blinded investigators [C.K., P.K. and W.K. (the latter as board‐certified dermatopathologist)] performed semiquantitative evaluation of all IHC stains, resulting in a consensus‐based score for each evaluation.

Based on previous studies,^11^ Ki‐67 immunostaining was graded as low (≤20% positive neoplastic cells, score 1) and strong (≥20%, score 2). The consensus‐based approach was particularly useful in tumours with a brisk immune infiltrate when distinction between Ki‐67‐positive dermal tumour cells and proliferating immune cells was necessary. This applied to 37 of 90 primary tumours (41.1% of all invasive melanomas).

Cyclin D1 immunostaining was evaluated regarding expression intensity and frequency of positive tumour cells separately and combined using a modified Allred score method^21^; scores (0) were graded as negative, (1–2) as weak, (3–5) as moderate and (6–8) as strong. Additionally, cyclin D1 and Ki‐67 expression were summed up as a composite score (1–4 low, 5–7 moderate, 8–10 strong). For further information, see Supporting information, Table S1.

### Statistical Analysis

Excel 2016 (Microsoft Corporation, Redmond, WA, USA) and spss for Windows version 25.0 (SPSS Inc., Chicago, IL, USA) were used for statistical analysis. Statistical significance was defined by a two‐sided *P*‐value of less than 0.05. The χ^2^ test was applied for comparisons between groups (MIS versus invasive melanoma, NMTM versus MTM) concerning nominally scaled variables, i.e. IHC expression intensities and patterns. Fisher's exact test was used, if applicable, for analyses of small subgroups. For comparisons concerning metric variables, either the *t*‐test or the Mann–Whitney *U*‐test were performed, depending on the assumed distribution of the respective variable. Multivariate analyses of potential prognostic factors were conducted through binary logistic regression. Pearson's bivariate analysis was conducted to assess potential correlations.

## Results

Data from 174 patients (65 MTM and 109 NMTM) were collected (see Supporting information, Figure S1). Of these, 52 (80%) and 94 (86.2%) blocks could be obtained, respectively. The final analysis comprised 42 (80.8%) samples in the MTM and 48 (51.1%) samples in the NMTM group, which remained after exclusion due to discrepant Breslow thickness or insufficient tissue material. In addition, 22 MIS samples were included for comparison between MIS and invasive melanoma.

### Patient and Tumour Characteristics

The study population consisted of 54 women and 58 men (see Table 1). Mean age at first diagnosis ranged from 49 to 72 years in the respective subgroups. The distribution of age between subgroups differed significantly (*P* < 0.001), as did the distribution of gender (*P = *0.032). Median Breslow thickness was 0.78 mm for MTM and 0.59 mm for NMTM (*P* < 0.001). Ulceration status was known in 94% of tumours. There was no significant difference between frequency of ulceration in tumours of the MTM (5%) and the NMTM (10%) subgroups (*P = *0.45).

**Table 1:** Patient and tumour characteristics

Clinical data				
	Metastatic (*n* = 42)	Non‐metastatic *n* = 48)	*In situ* (*n* = 22)	*P* M/NMTM
AJCC stage IV	28 (66.7%)			
AJCC stage III	14 (33.3%)			
Female	15 (35.7%)	28 (58.3%)	11 (50%)	0.032^†^
Male	27 (64.3%)	20 (41.7%)	11 (50%)	
Mean age at first diagnosis (years)	49.2 (95% CI = 44.8–53.7)	62.9 (95% CI = 58.2–67.5)	71.5 (95% CI = 65–78)	< 0.001*
Breslow (mm)				
Mean	0.78 95% CI = 0.72–0.84)	0.59 (95% CI = 0.55–0.63)		<0.001^‡^
Median	0.80 95% CI = 0.74–0.86)	0.58 (95% CI = 0.53–0.62)		
Histological subtype				
SSM	24 (57.1%)	20 (41.7%)	11 (50%)	Invasive
NMM	0	2 (4.2%)	0	0.22^†^
ALM	3 (7.1%)	3 (6.2%)	0	
LMM	1 (2.4%)	6 (12.5%)	11 (50%)	Invasive/MIS
Others/unknown	14 (33.4%)	17 (35.4%)	0	<0.001^†^
Anatomical site				
Head/neck	7 (16.7%)	7 (14.6%)	1 (4.6%)	0.44^†^
Upper extremities	4 (9.5%)	11 (22.9%)	1 (4.6%)	
Lower extremities	12 (28.6%)	15 (31.2%)	1 (4.6%)	
Trunk	17 (35.7%)	12 (25%)	2 (9%)	
Acra	4 (9.5%)	3 (6.3%)	0	
Unknown	0	0	17 (77.2%)	
Mitoses/mm^2^				
<1	31 (73.8%)	39 (81.3%)		0.4^†^
≥1	11 (26.2%)	9 (18.7%)		
Ulceration				
Present	2 (4.8%)	5 (10.4%)		0.45^†^
Absent	37 (88.1%)	41 (85.4%)		
Unknown	3 (7.1%)	2 (4.2%)		
SLNB				
Positive	9 (21.4%)	0		0.017^†^
Negative	7 (16.7%)	6 (12.5%)		
Not performed	26 (61.9%)	42 (87.5%)		
Marked inflammation				
Present	17 (40.5%)	20 (41.7%)		
Absent	25 (59.5%)	28 (58.3%)		

SSM, Superficial spreading melanoma; NMM, Nodular melanoma; MIS, melanoma *in‐situ*; ALM, Acral lentiginous melanoma; LMM, Lentigo maligna melanoma; AJCC, American Joint Committee on Cancer; NMTM, Non‐metastatic; MTM, metastatic.

* *t*‐test,

^†^ χ^2^ test,

^‡^ Mann–Whitney *U*‐test.

The predominant histological subtype of invasive melanoma was superficial spreading melanoma (SSM) in 55 of 112 samples (49.1%), representing 57.1% of the MTM and 43.8% of the NMTM groups. In MIS patients, SSM and lentigo maligna melanoma (LMM) equalled 45.5% (10 of 22); 35.7% of all MTMs were localised on the trunk, whereas NMTMs were most frequently detected on the lower extremity (31.2%).

We did not detect a difference in mitotic rate (MR, documented as < 1 or ≥ 1 mitoses per mm^2^) comparing invasive MTM and NMTM. Increased MR was found in 11 of 42 patients (26%) in the MTM and in nine of 48 patients (19%, *P* = 0.4) in the NMTM groups, respectively.

Sentinel lymph node biopsy (SLNB) was performed in a total of 22 of 90 patients (24.4%) with invasive melanoma. SLNB was conducted in 38% of patients with MTM and 12.5% of patients with NMTM. In MTM patients, the SLNB result was positive in nine patients (56%), while no patient in the NMTM group had a positive SLN (which would have been an exclusion criterion for this subgroup). Additional tumour characteristics of MTM and NMTM according to Breslow thickness can be found in Supporting information, Tables S2–S5.

### Immunohistochemistry Findings

The results of IHC findings in MIS, NMTM and MTM are summarised in Table 2.

**Table 2:** Histological data

Histological data					
	Metastatic (*n* = 42)	Non‐metastatic (*n* = 48)	*In situ* (*n* = 22)	*In situ* versus invasive*	Non‐metastatic versus metastatic
*Cyclin D1 intensity*					
None (0)	2 (4.8%)	0	0		*P* = 0.025
Weak (1)	8 (19%)	9 (18.8%)	9 (40.9%)		
Intermediate (2)	20 (47.6%)	16 (33.3%)	11 (50%)		
Strong (3)	12 (28.6%)	23 (47.9%)	2 (9.1%)		
*Cyclin D1‐positive cells epidermal*					
0	1 (2.4%)	0	0	*P* = 0.003	*P* = 0.611
1%	2 (4.8%)	1 (2.1%)	1 (4.6%)		
1–10%	7 (16.7%)	10 (20.8%)	14 (63.5%)		
10–33%	19 (45.1%)	21 (43.8%)	5 (22.7%)		
33–66%	7 (16.7%)	12 (25%)	2 (9.2%)		
> 66%	4 (9.5%)	4 (8.3%)	0		
NA	2 (4.8%)	0	0		
*Cyclin D1‐positive cells dermal*					
0	9 (21.4%)	10 (20.8%)			*P* = 0.883
1%	4 (9.5%)	6 (12.6%)			
1–10%	11 (26.2%)	16 (33.3%)			
10–33%	9 (21.4%)	10 (20.8 %)			
33–66%	3 (7.2%)	2 (4.2 %)			
> 66%	0	0			
NA	6 (14.3%)	4 (8.3%)			
*Allred score epidermal*					
None (0)	1 (2.4%)	0	0		*P* = 0.588
Weak (1–2)	1 (2.4%)	1 (2.1%)	1 (4.6%)		
Intermediate (3–5)	24 (57.2%)	22 (45.8%)	18 (81.8%)		
Strong (6–8)	14 (33.2%)	25 (52.1%)	3 (13.6%)		
NA	2 (4.8%)	0	0		
*Allred score dermal*					
None (0)	1 (2.4%)	0			*P* = 0.502
Weak (1–2)	11 (26.2%)	8 (16.7%)			
Intermediate (3–5)	17 (40.4%)	27 (56.2%)			
Strong (6–8)	7 (16.7%)	9 (18.8%)			
NA	6 (14.3%)	4 (8.3%)			
*Ki‐67‐positive cells epidermal*					
≤20%	13 (30.9%)	23 (47.9%)	10 (45.4%)	*P* = 0.699	*P* = 0.148
≥20%	27 (64.3%)	25 (52.1%)	12 (54.6%)		
NA	2 (4.8%)	0	0		
*Ki‐67 positive‐cells dermal*					
≤20%	18 (42.8%)	38 (79.1%)			*P* = 0.001
≥20%	12 (28.6%)	4 (8.3%)			
NA	12 (28.6%)	6 (12.6%)			
*Ki‐67 + cyclin D1 epidermal*					
Low (0–4)	13 (31%)	20 (41.7%)	21 (95.4%)		*P* = 0.386
High (5–7)	25 (59.5%)	28 (58.3%)	1 (4.6%)		
NA	4 (9.5)	0	0		
*Ki‐67 + cyclin D1 dermal*					
Low (0–4)	19 (45.2%)	38 (79.2%)			*P* = 0.003
High (5–7)	8 (19.1%)	1 (2.1%)			
NA	15 (35.7%)	9 (18.7%)			

* Invasive = non‐metastatic (NMTM) and metastatic (MTM) grouped together.

**Figure 1:**
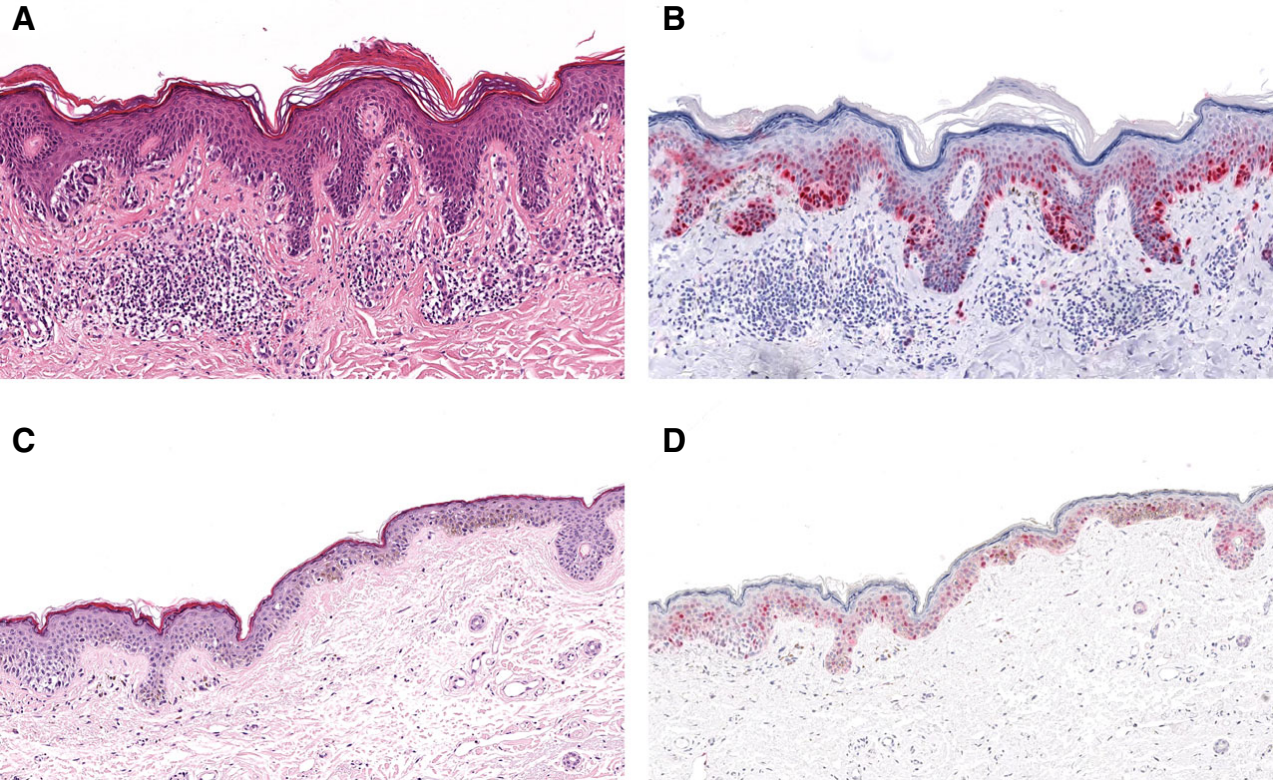
Illustration of increased epidermal cyclin D1 expression in thin invasive melanoma (**A**,**B**) compared to *in‐situ* melanoma (**C**,**D**). **A**,**C**, haematoxylin and eosin stain; **B**,**D**, cyclin D1 stain.

First, epidermal expression of Ki‐67 and cyclin D1 in MIS and invasive melanoma (MTM and NMTM grouped together) was analysed. No statistically significant difference in Ki‐67 expression was observed between groups (*P = *0.7). In contrast, a significantly higher percentage of cyclin D1‐positive tumour cells was found in thin invasive melanoma compared to MIS (*P = *0.003, Figure 1).

**Figure 2:**
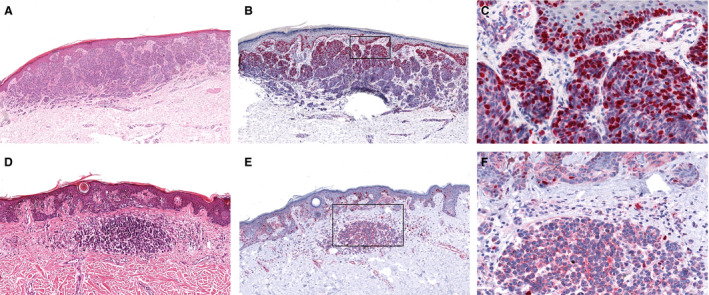
Increased frequencies of dermal Ki‐67 positive tumour cells were found in metastatic (**A**–**C**) compared to non‐metastatic (**D**–**F**) thin melanomas (*P* = 0.001).

Secondly, epidermal and dermal expression in NMTM and MTM were compared. In the epidermis, no difference was detected between both groups (*P = *0.15 for Ki‐67; *P = *0.61 for cyclin D1). Cyclin D1 expression in the dermis was similar in both groups (*P = *0.88). Conversely, there was a significantly higher percentage of strong dermal Ki‐67 expression in MTM compared to NMTM (28.6 and 8.3%; *P = *0.001, Figure 2).

Analysing dermal Ki‐67 expression together with the established prognostic parameter mitotic rate did not alter this finding significantly *(P = *0.003). The prognostic value of epidermal Ki‐67 expression was retained (*P = *0.047) when other prognostic factors (Breslow thickness) and potential confounders (age, gender) – which were significantly different between groups – were taken into account in a multivariate analysis. There was no correlation between Breslow thickness and dermal Ki‐67 expression neither overall (Pearson's *r = *0.17, *P = *0.14), nor within the MTM subgroup (Pearson's *r = –*0.14, *P = *0.46).

When analysing melanoma subtypes, epidermal cyclin D1 expression appeared to be increased in SSM compared to LMM in univariate analysis (*P = *0.02). Also, there was a trend towards increased epidermal cyclin D1 expression in the small subgroup of acral lentiginous melanoma (ALM, six patients) compared to both SSM (*P = *0.07) and LMM (*P = *0.07); see Table 3.

**Table 3:** Epidermal cyclin D1 expression in different subtypes

Epidermal cyclin D1 expression in different subtypes										
								*P* ^†^		
Cyclin D1‐positive cells		0	1%	1–10%	10–33%	33–66%	> 66%	SSM/LMM	SSM/ALM	LMM/ALM
SSM	55 (49.1%)	3 (5.5%)	2 (3.6%)	5 (9.1%)	31 (56.4%)	10 (18.2%)	4 (7.3%)	0.019	0.015	0.059
LMM	18 (16.1%)	1 (5.6%)	1 (5.6%)	8 (44.4%)	5 (27.8%)	3 (16.7%)	0			
ALM	6 (5.4%)	0	1 (16.7%)	1 (16.7%)	0	2 (33.3%)	2 (33.3%)			
NM	2 (1.8%)									
Other	31 (27.7%)									

SSM, Superficial spreading melanoma; LMM, Lentigo maligna melanoma; ALM, Acral lentiginous melanoma; NM, Non‐metastatic.

^†^ Fisher's exact test.

### Marginal Note: Breslow Measurement

Breslow thickness was remeasured for every sample obtained. As described above, seven of 174 specimens (4% of the total of MTM and NMTM) had to be excluded due to initial underestimation of tumour thickness. Median mismeasurement was 0.75 mm [95% confidence interval (CI)* = *0.55–0.95).

In our study cohort (MTM and NMTM only, *n = *90), documented Breslow thickness was higher than the remeasured value in 32, lower in 36 and equal in 22 samples (35.6, 40 and 24.4%, respectively). Median discrepancy was 0.10 mm (95% CI* = *0.07–0.13) and 0.14 mm (95% CI* = *0.10–0.18).

## Discussion

Thin melanomas less than 1 mm in Breslow index rarely metastasise but, owing to their high incidence, still lead to a higher overall number of melanoma‐related deaths than high‐risk melanomas 4 mm or more in thickness.^2^, ^3^ The median duration between first melanoma diagnosis and death appears to be significantly longer in patients with thin primaries compared to those with thicker tumours.^3^ This may be explained by the more aggressive biology of thicker melanomas, but may also result from later diagnosis of metastasis in patients with thin primaries. Late diagnosis of metastasis could, in turn, be the consequence of less stringent follow‐up protocols in so‐called low‐risk patients with thin melanoma. As more rigorous follow‐up does not seem justifiable in this patient population, the discovery of biomarkers predictive of metastasis in thin melanoma is of utmost importance. The present study aimed to re‐evaluate the predictive potential of two such potential biomarkers; namely, Ki‐67 and cyclin D1.

Our findings suggest that high dermal Ki‐67 expression is an independent prognostic marker associated with an increased risk of metastasis formation in thin melanoma. This is in line with a large study by Gimotty *et al*.,^11^ who also reported dermal but not overall Ki‐67 expression to be an independent prognostic marker in a cohort of 396 patients with more than 10 years of follow‐up. Despite its significance, there are certain caveats for the use of Ki‐67 in clinical practice. As mentioned in the Methods section, it is frequently difficult to distinguish proliferating dermal tumour cells from proliferating immune cells i.e., tumour‐infiltrating lymphocytes, in single‐antibody Ki‐67 IHC. Hence, double‐staining IHC with a melanocytic differentiation marker such as Melan‐A or MART‐1 could increase sensitivity and specificity of Ki‐67 staining. Combination with methods such as gene expression signatures could further augment the prognostic value,^22^ although the validity of such signatures remains to be confirmed in larger prospective studies.

Gimotty and others^11^, ^23^, ^24^, ^25^ also reported on the prognostic potential of increased mitotic rate in thin melanoma, which previously was also included in the AJCC staging system.^20^ In our cohort of thin melanomas, we could not confirm the prognostic potential of mitotic rate, as increased mitoses were rare in both the metastatic and non‐metastatic subgroups.

Investigating the stepwise development of melanoma from *in‐situ* to invasive lesions, we found statistically significant differences in epidermal cyclin D1 expression between *in‐situ* and invasive melanomas. These results contrast with some studies on cyclin D1, which reported weak expression in more than two‐thirds of melanoma samples and no expression in naevi, respectively, but are in accordance with other reports describing overall high expression in melanocytic lesions.^16^, ^26^, ^27^ Ramirez *et al*. evaluated 126 pigmented skin lesions (of 28 *in‐situ*, 30 primary invasive and 29 metastatic melanomas). They found a higher rate of cyclin D1 expression in invasive and *in‐situ* melanoma compared with benign melanocytic lesions. Contrary to our findings, these authors found an increased cyclin D1 expression in melanoma *in situ* compared to invasive melanoma, which could be explained by the higher number of melanomas of more than 1 mm Breslow thickness in this study, as the latter tumours showed a trend towards decreased cyclin D1 expression.^28^

The proto‐oncogenic role of cyclin D1 has been characterised in various studies.^16^, ^17^, ^18^, ^19^ We propose that our immunohistochemical finding of an increased cyclin D1 expression in thin invasive melanoma also suggests a potential role of cyclin D1 during the process of invasion in the stepwise evolution of melanoma, as extensively described by Shain *et al*.^28^, ^29^

Alongside cyclin D1, genetic alterations leading to acquisition of invasive behaviour have been comprehensively investigated. Loss of the *cyclin‐dependent kinase inhibitor 2A* (*CDKN2A*) tumour suppressor gene is the most common acquired genetic change in invasive melanoma.^5^ Bastian *et al*.^30^ described the mechanism of melanoma initiation as a result of bi‐allelic loss of *CDKN2A*, and consequently p16^INK4A^, via activation of BRN2, a transcription factor downstream of *CDKN2A*. Further investigations will be necessary to fully elucidate all aspects of invasion, thereby possibly uncovering new prognostic markers.

Inevitably, the results of our study need to be interpreted with certain limitations in mind. Matching of the two main groups (MTM and NMTM) was achieved for most characteristics, except for age, subtype and Breslow thickness. Exclusion of approximately 50% of all samples after acquisition from external laboratories for the above‐mentioned reasons resulted in this unfortunate imbalance of subgroups regarding certain aspects. However, and most importantly, we believe that the difference in Breslow thickness between the MTM and NMTM group does not represent a significant confounder of our main findings for two reasons. First, dermal Ki‐67 expression also retained its prognostic value when Breslow thickness was taken into account in a multivariate analysis. Secondly, we did not detect any correlation between dermal Ki‐67 expression and Breslow thickness, particularly not in the MTM subgroup in which median Breslow was increased.

In this context, discrepancy in Breslow measurement has to be addressed briefly. Our values for tumour thickness differed in a surprising number of patients when external samples were re‐evaluated. This is particularly of importance if the mismeasurement leads to ‘understaging’ of disease. These findings underline that a multi‐eye principle is essential and generally advisable in melanoma histopathology.

In conclusion, we found an association of dermal Ki‐67 expression with an increased risk of metastasis development in thin primary melanomas. The usefulness of Ki‐67 expression as a prognostic marker should be considered in clinical practice and could be enhanced through combination with other methods. Although our findings suggest a potential role of cyclin D1 during early invasion of melanoma cells, expression of this protein alone does not appear to be of prognostic significance.

## Conflicts of interest

None declared.

## Supporting information


**Figure S1.** Flow chart of sample acquisition.

**Table S1.** Grading syste.
**Table S2.** MTM, Patient and lesion characteristics according to Breslow thickne.
**Table S3.** MTM, IHC data according to Breslow thickness.
**Table S4.** NMTM, Patient and lesion characteristics according to Breslow thickne.
**Table S5.** NMTM, IHC data according to Breslow thickness thickne.
**Table S6.** Antibodies and Staining Protocol.
