# Supplementary figures and images for: The role of cyclin D1 and Ki‐67 in the development and prognostication of thin melanoma
Source: Histopathology. 2020 Jul 4;77(3):460–70. doi: 10.1111/his.14139 (PMC7540531; doi:10.1111/his.14139)

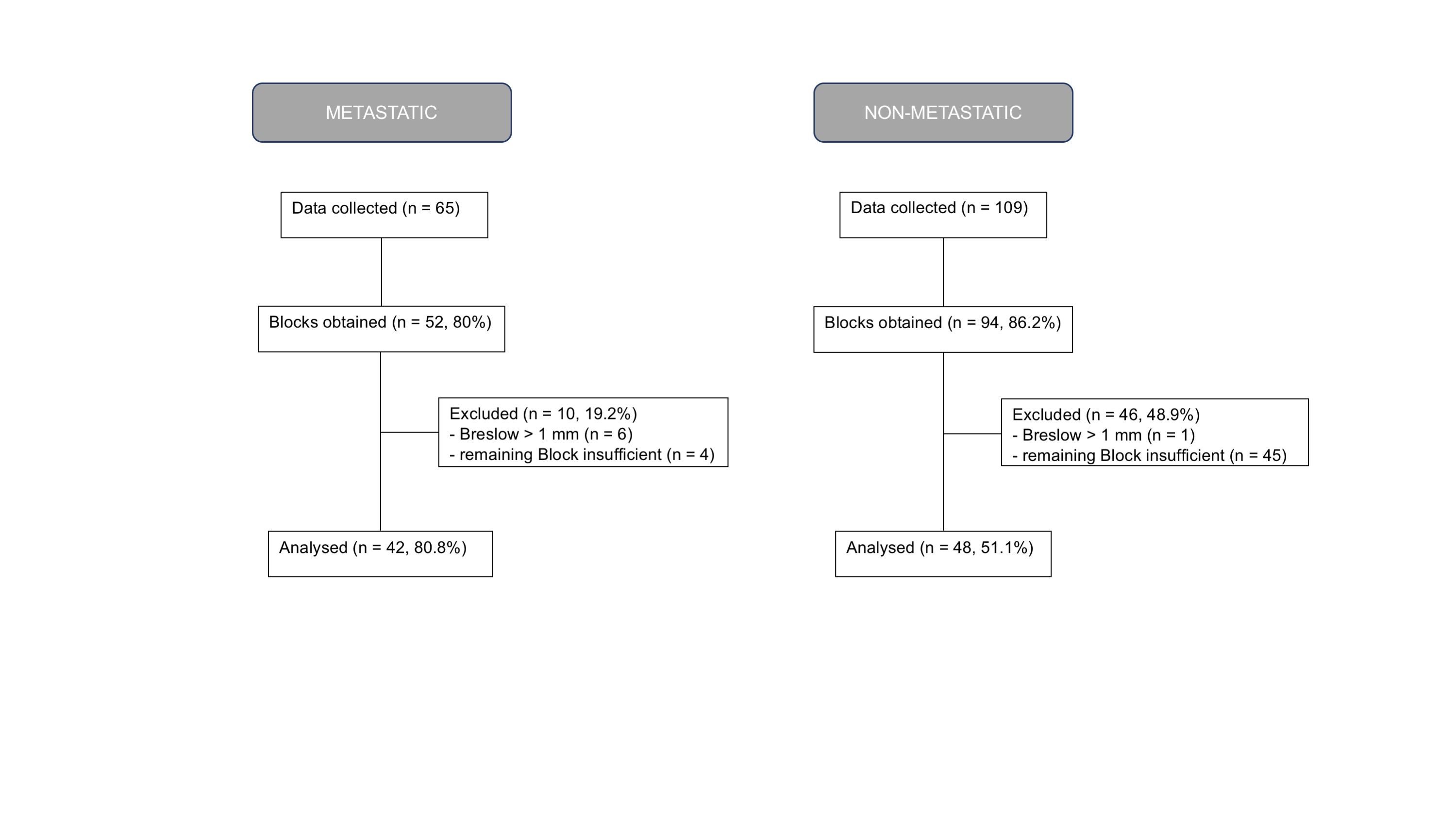

Supplement: Supplementary file 1 — Figure S1. Flow chart of sample acquisition. [file HIS-77-460-s001.jpg]
