# Supplementary material for: The role of cyclin D1 and Ki‐67 in the development and prognostication of thin melanoma
Source: Histopathology. 2020 Jul 4;77(3):460–70. doi: 10.1111/his.14139 (PMC7540531; doi:10.1111/his.14139)
Supplement: Supplementary file 2 — Table S1. Grading syste. Table S2. MTM, Patient and lesion characteristics according to Breslow thickne. Table S3. MTM, IHC data according to Breslow thickness. Table S4. NMTM, Patient and lesion characteristics according to Breslow thickne. Table S5. NMTM, IHC data according to Breslow thickness thickne. Table S6. Antibodies and Staining Protocol. [file HIS-77-460-s002.docx]

**Supplementary Table 1:** Grading system

| ***Grading system*** | | | | | | | | |
| --- | --- | --- | --- | --- | --- | --- | --- | --- |
| Ki-67 positive cells | 1: ≤ 20% | | | | 2: ≥ 20% | | | |
| Cyclin D1 positive cells | 0: 0% | 1: 1% | | 2: 1 - 10% | 3: 10 - 33% | 4: 33 - 66% | | 5: ≥ 66% |
| Cyclin D1 staining intensity | 0: none | | 1: weak | | 2: intermediate | | 3: strong | |
| Modified Allred score: Cyclin D1 positive cells + Cyclin D1 intensity | 0: negative | | 1 - 2: weak | | 3 - 5: moderate | | 6 - 8: strong | |
| Ki-67 + Cyclin D1 positive cells | 0 - 4: low | | | | 5 - 7: high | | | |

**Supplementary Table 2:** MTM, Patient and lesion characteristics according to Breslow thickness

| ***Metastatic thin melanomas: Patient and lesion characteristics according to Breslow thickness (n*** ***=*** ***42)*** | | | | | | | | | |
| --- | --- | --- | --- | --- | --- | --- | --- | --- | --- |
| mm | | | ≤ 0.25 | 0.26 - 0.5 | | 0.51 - 0.74 | | 0.75 - 1.0 | |
|  | | | 0 | 6 (14.3%) | | 12 (28.6%) | | 24 (57.1%) | |
| Breslow [mm]  Mean  Median | | |  | 0.48 (95% CI = 0.43 - 0.52)  0.50  (95% CI = 0.46 - 0.54) | | 0.66  (95% CI = 0.62 - 0.70)  0.70  (95% CI = 0.66 - 0.74) | | 0.91  (95% CI = 0.86 - 0.96)  0.90  (95% CI = 0.85 - 0.95) | |
| Mean Age [y] | | |  | 54.1  (95% CI = 40.9 - 67.3) | | 52.7  (95% CI = 43.9 - 61.6) | | 46.2  (95% CI = 40 - 52.5) | |
| Sex | f | m |  | 0 | 6 (100%) | 4  (33.3%) | 8  (66.7%) | 11 (45.8%) | 13 (54.2%) |
| *Subtype* | | | | | | | | | |
| SSM | | |  | 3 (50%) | | 6 (50%) | | 15 (62.5%) | |
| LMM | | |  | 0 | | 1 (8.3%) | | 0 | |
| ALM | | |  | 1 (16.7%) | | 1 (8.3%) | | 1 (4.2%) | |
| NM | | |  | 0 | | 0 | | 0 | |
| Rare / unknown | | |  | 2 (33.3%) | | 4 (33.4%) | | 8 (33.3%) | |
| *Localisation* | | | | | | | | | |
| Head / neck | | |  | 0 | | 3 (25%) | | 4 (16.7%) | |
| Upper limb | | |  | 1 (16.7%) | | 2 (16.7%) | | 1 (4.2%) | |
| Lower limb | | |  | 3 (50%) | | 4 (33.3%) | | 5 (20.7%) | |
| Acral | | |  | 1 (16.7%) | | 2 (16.7%) | | 1 (4.2%) | |
| Trunk | | |  | 1 (16.7%) | | 1 (8.3%) | | 13 (54.2%) | |
| *Mitoses / mm^2^* | | | | | | | | | |
| < 1 | | |  | 6 (100%) | | 10 (83.3%) | | 15 (62.5%) | |
| ≥ 1 | | |  | 0 | | 2 (16.7%) | | 9 (37.5%) | |
| *Ulceration* | | | | | | | | | |
| Present | | |  | 2 (33.3%) | | 0 | | 2 (8.3%) | |
| Absent | | |  | 1 (16.7%) | | 12 (100%) | | 22 (91.7%) | |
| Unknown | | |  | 3 (50%) | | 0 | | 0 | |
| *SLNB* | | | | | | | | | |
| positive | | |  | 1 (16.7%) | | 0 | | 8 (33.3%) | |
| negative | | |  | 1 (16.7%) | | 3 (25%) | | 3 (12.5%) | |
| Not performed | | |  | 4 (66.7%) | | 9 (75%) | | 13 (54.2%) | |

**Supplementary Table 3:** MTM, IHC data according to Breslow thickness

| ***Metastatic thin melanomas: IHC data according to Breslow thickness (n*** ***=*** ***42*)** | | | | |
| --- | --- | --- | --- | --- |
| mm | ≤ 0.25 | 0.26 - 0.5 | 0.51 - 0.74 | 0.75 - 1.0 |
|  | 0 | 6 (14.3%) | 12 (28.6%) | 24 (57.1%) |
| *Cyclin D1 intensity* | | | | |
| None (0) |  | 0 | 1 (8.3%) | 1 (4.2%) |
| Weak (1) |  | 0 | 3 (25%) | 5 (20.7%) |
| Intermediate (2) |  | 4 (66.7%) | 5 (41.7%) | 11 (45.8%) |
| Strong (3) |  | 2 (33.3%) | 3 (25%) | 7 (29.3%) |
| *Cyclin D1 positive cells epidermal* | | | | |
| 0 |  | 0 | 0 | 1 (4.2%) |
| 1% |  | 0 | 0 | 2 (8.3%) |
| 1 - 10% |  | 1 (16.7%) | 2 (16.7%) | 4 (16.6%) |
| 10 - 33% |  | 4 (66.7%) | 6 (50%) | 9 (37.4%) |
| 33 - 66% |  | 1 (16.7%) | 2 (16.7%) | 4 (16.7%) |
| > 66% |  | 0 | 2 (16.7%) | 2 (8.4%) |
| NA |  | 0 | 0 | 2 (8.4%) |
| *Cyclin D1 positive cells dermal* | | | | |
| 0 |  | 3 (50%) | 2 (16.7%) | 4 (16.7%) |
| 1% |  | 0 | 1 (8.3%) | 3 (12.5%) |
| 1 - 10% |  | 2 (33.3%) | 2 (16.7%) | 7 (29.2%) |
| 10 - 33% |  | 0 | 3 (25%) | 6 (25%) |
| 33 - 66% |  | 0 | 1 (8.3%) | 2 (8.3%) |
| > 66% |  | 0 | 0 | 0 |
| NA |  | 1 (16.7%) | 3 (25%) | 2 (8.3%) |
| *Ki-67 positive cells epidermal* | | | | |
| ≤ 20% |  | 3 (50%) | 9 (75%) | 7 (29.2%) |
| ≥ 20% |  | 3 (50%) | 3 (25%) | 15 (62.5%) |
| NA |  | 0 | 0 | 2 (8.3%) |
| *Ki-67 positive cells dermal* | | | | |
| ≤ 20% |  | 1 (16.7%) | 5 (41.7%) | 12 (50%) |
| ≥ 20% |  | 1 (16.7%) | 4 (33.3%) | 7 (29.1%) |
| NA |  | 4 (66.6%) | 3 (25%) | 5 (20.9%) |

**Supplementary Table 4:** NMTM, Patient and lesion characteristics according to Breslow thickness

| ***Non-metastatic thin melanomas: Patient and tumour characteristics according to Breslow thickness (n*** ***=*** ***48)*** | | | | | | | | | | | | |
| --- | --- | --- | --- | --- | --- | --- | --- | --- | --- | --- | --- | --- |
| mm | | | ≤ 0.25 | | 0.26 - 0.5 | | | 0.51- 0.74 | | | 0.75 - 1.0 | |
|  | | | 1 (2.1%) | | 13 (27.1%) | | | 27 (56.2%) | | | 7 (14.6%) | |
| Breslow [mm]  Mean  Median | | | 0.25 | | 0.43  (95% CI = 0.39 - 0.48)  0.45  (95% CI = 0.41 - 0.49) | | | 0.62  (95% CI = 0.59 - 0.64)  0.60  (95% CI = 0.57 - 0.63) | | | 0.82  (95% CI = 0.75 - 0.89)  0.80  (95% CI = 0.73 - 0.87) | |
| Mean Age [years] | | | 82 | | 64.7  (95% CI = 52.4 - 77.1) | | | 59.4  (95% CI = 53.8 - 64.9) | | | 70.2  (95% CI = 58.4 - 81.9) | |
| Sex | f | m | 1  (100%) | 0 | 7  (53.9%) | 6  (46.1%) | | 18 (66.7%) | 9  (33.3%) | | 2  (28.6%) | 5  (71.4%) |
| *Subtype* | | | | | | | | | | | | |
| SSM | | | 0 | | 5 (38.5%) | | 12 (44.4%) | | | 3 (42.8%) | | |
| LMM | | | 1 (100%) | | 3 (23.1%) | | 2 (7.4%) | | | 0 | | |
| ALM | | | 0 | | 1 (7.7%) | | 1 (3.7%) | | | 1 (14.3%) | | |
| NM | | | 0 | | 0 | | 1 (3.7%) | | | 1 (14.3%) | | |
| Rare / unknown | | | 0 | | 4 (30.7%) | | 11 (40.8%) | | | 2 (28.6%) | | |
| *Localisation* | | | | | | | | | | | | |
| Head / neck | | | 1 (100%) | | 1 (7.7%) | | 3 (11.1%) | | | 2 (28.6%) | | |
| Upper limb | | | 0 | | 5 (38.5%) | | 5 (18.6%) | | | 1 (14.3%) | | |
| Lower limb | | | 0 | | 3 (23.1%) | | 10 (37%) | | | 2 (28.6%) | | |
| Acral | | | 0 | | 0 | | 3 (11.1%) | | | 0 | | |
| Trunk | | | 0 | | 4 (30.7%) | | 6 (22.2%) | | | 2 (28.6%) | | |
| *Mitoses / mm^2^* | | | | | | | | | | | | |
| < 1 | | | 1 (100%) | | 12 (92.3%) | | 20 (74.1%) | | | 6 (85.7%) | | |
| ≥ 1 | | | 0 | | 1 (7.7%) | | 7 (24.9%) | | | 1 (14.3%) | | |
| *Ulceration* | | | | | | | | | | | | |
| Present | | | 0 | | 0 | | 3 (11.1%) | | | 2 (28.6%) | | |
| Absent | | | 1 (100%) | | 11 (84.6%) | | 24 (88.9%) | | | 5 (61.4%) | | |
| Unknown | | | 0 | | 2 (15.4%) | |  | | |  | | |
| *SLNB* | | | | | | | | | | | | |
| positive | | | 0 | | 0 | | 0 | | | 0 | | |
| negative | | | 0 | | 0 | | 3 (11.1%) | | | 3 (42.8%) | | |
| Not performed | | | 1 (100%) | | 13 (100%) | | 24 (88.9%) | | | 4 (57.2%) | | |

**Supplementary Table 5:** NMTM, IHC data according to Breslow thickness thickness

| ***Non-metastatic thin melanomas: IHC data according to Breslow thickness (n=48)*** | | | | |
| --- | --- | --- | --- | --- |
| mm | ≤ 0.25 | 0.26 - 0.5 | 0.51 - 0.74 | 0.75 - 1.0 |
|  | 1 (2.1%) | 13 (27.1%) | 27 (56.2%) | 7 (14.6%) |
| *Cyclin D1 intensity* | | | | |
| None (0) | 0 | 0 | 0 | 0 |
| Weak (1) | 0 | 2 (15.4%) | 7 (25.9%) | 0 |
| Intermediate (2) | 0 | 5 (38.5%) | 9 (33.3%) | 2 (28.6%) |
| Strong (3) | 1 (100%) | 6 (46.1%) | 11 (40.8%) | 5 (71.4%) |
| *Cyclin D1 positive cells epidermal* | | | | |
| 0 | 0 | 0 | 0 | 0 |
| 1% | 0 | 1 (7.7%) | 0 | 0 |
| 1 - 10% | 0 | 2 (15.4%) | 7 (25.9%) | 1 (14.3%) |
| 10 - 33% | 0 | 3 (23.1%) | 14 (51.9%) | 4 (57.1%) |
| 33 - 66% | 1 (100%) | 6 (46.1%) | 4 (14.8%) | 1 (14.3%) |
| > 66% | 0 | 1 (7.7%) | 2 (7.4%) | 1 (14.3%) |
| NA | 0 | 0 | 0 | 0 |
| *Cyclin D1 positive cells dermal* | | | | |
| 0 | 0 | 4 (30.8%) | 5 (18.6%) | 1 (14.3%) |
| 1% | 0 | 1 (7.7%) | 5 (18.6%) | 0 |
| 1 - 10% | 1 (100%) | 0 | 13 (48.1%) | 2 (28.6%) |
| 10 - 33% | 0 | 4 (30.7%) | 3 (11.1%) | 3 (42.8%) |
| 33 - 66% | 0 | 2 (15.4%) | 0 | 0 |
| > 66% | 0 | 0 | 0 | 0 |
| NA | 0 | 2 (15.4%) | 1 (3.6%) | 1 (14.3%) |
| *Ki-67 positive cells epidermal* | | | | |
| ≤ 20% | 1 (100%) | 7 (53.8%) | 13 (48.1%) | 2 (28.6%) |
| ≥ 20% | 0 | 6 (46.2%) | 14 (51.9%) | 5 (71.4%) |
| NA | 0 | 0 | 0 | 0 |
| *Ki-67 positive cells dermal* | | | | |
| ≤ 20% | 1 (100%) | 11 (84.6%) | 19 (70.4%) | 7 (100%) |
| ≥ 20% | 0 | 0 | 4 (14.8%) | 0 |
| NA | 0 | 2 (15.4%) | 4 (14.8%) | 0 |

**Supplementary Table 6:** Antibodies and Staining Protocol
